# Establishing a Highly Accurate Circulating Tumor Cell Image Recognition System for Human Lung Cancer by Pre-Training on Lung Cancer Cell Lines

**DOI:** 10.3390/cancers17142289

**Published:** 2025-07-09

**Authors:** Hiroki Matsumiya, Kenji Terabayashi, Yusuke Kishi, Yuki Yoshino, Masataka Mori, Masatoshi Kanayama, Rintaro Oyama, Yukiko Nemoto, Natsumasa Nishizawa, Yohei Honda, Taiji Kuwata, Masaru Takenaka, Yasuhiro Chikaishi, Kazue Yoneda, Koji Kuroda, Takashi Ohnaga, Tohru Sasaki, Fumihiro Tanaka

**Affiliations:** 1 Second Department of Surgery, School of Medicine, University of Occupational and Environmental Health, Kitakyushu 807-8555, Japan; masataka-m@med.uoeh-u.ac.jp (M.M.); masatoshi-kanayama@med.uoeh-u.ac.jp (M.K.); rintarooyama@med.uoeh-u.ac.jp (R.O.); yukikofukuichi@med.uoeh-u.ac.jp (Y.N.); nmasamed@med.uoeh-u.ac.jp (N.N.); yoheihonda@med.uoeh-u.ac.jp (Y.H.); t-kuwata@med.uoeh-u.ac.jp (T.K.); m-takenaka@med.uoeh-u.ac.jp (M.T.); cywmd0k2@med.uoeh-u.ac.jp (Y.C.); yoneda@med.uoeh-u.ac.jp (K.Y.); kuroda-k@med.uoeh-u.ac.jp (K.K.); ohnaga@pe.ctt.ne.jp (T.O.); ftanaka@med.uoeh-u.ac.jp (F.T.); 2 Department of Mechanical and Intellectual Systems Engineering, Faculty of Engineering, University of Toyama, Toyama 930-8555, Japan; tera@eng.u-toyama.ac.jp (K.T.); yusuke.kishi1@konicaminolta.com (Y.K.); y.yoshino@shimz.co.jp (Y.Y.); tsasaki@eng.u-toyama.ac.jp (T.S.)

**Keywords:** lung cancer, tumor cell, artificial intelligence, cell lines, transfer learning

## Abstract

Background/Objectives: Circulating tumor cells (CTCs) are important biomarkers for predicting prognosis and evaluating treatment efficacy in cancer. We developed the “CTC-Chip” system based on microfluidics, enabling highly sensitive CTC detection and prognostic assessment in lung cancer and malignant pleural mesothelioma. However, the final identification and enumeration of CTCs require manual intervention, which is time-consuming, prone to human error, and necessitates the involvement of experienced medical professionals. Medical image recognition using machine learning can reduce workload and improve automation. However, CTCs are rare in clinical samples, limiting the training data available to construct a robust CTC image recognition system. In this study, we established a highly accurate artificial intelligence-based CTC recognition system by pre-training convolutional neural networks using images from lung cancer cell lines. Methods: We performed transfer learning of convolutional neural networks. Initially, the models were pre-trained using images obtained from lung cancer cell lines. The model’s accuracy was improved by training with a limited number of clinical CTC images. Results: Transfer learning significantly improved the CTC classification accuracy to an average of 99.51%, compared to 96.96% for a model trained solely on pre-trained cell lines (*p* < 0.05). This approach showed notable efficacy when clinical training images were limited, achieving statistically significant accuracy improvements with as few as 17 clinical CTC images (*p* < 0.05). Conclusions: Overall, our findings demonstrate that pre-training with cancer cell lines enables rapid and highly accurate automated CTC recognition even with limited clinical data, significantly enhancing clinical applicability and potential utility across diverse cancer diagnostic workflows.

## Simple Summary

Circulating tumor cells (CTCs) are rare cancer cells in the blood that can help predict treatment outcomes. However, identifying them manually is slow and needs expertise. In this study, we developed an AI system that accurately detects CTCs using image recognition. To solve the problem of limited clinical images, we first trained the AI system with lung cancer cell line images and then applied transfer learning using a small number of real CTC images. This approach significantly improved accuracy, even with only 17 clinical images. The final model reached 99.5% accuracy. This method reduces the need for large clinical datasets and supports faster, more reliable CTC detection in lung cancer. It may also be applicable to other cancer types and diagnostic workflows.

## 1. Introduction

Circulating tumor cells (CTCs) are indicators of cancer micrometastases and are clinically useful for prognostic prediction and treatment monitoring [1,2,3,4,5,6,7,8,9]. The CellSearch system is a semi-automated system for quantitative CTC evaluation demonstrating high reproducibility and clinical utility for monitoring metastatic breast, colorectal, and prostate cancers [10,11]. However, CellSearch lacks sensitivity for detecting micrometastases in lung cancer [12]. To address this limitation, we developed a highly sensitive microfluidic-based CTC detection system, known as the “CTC-Chip,” which primarily utilizes antigen–antibody interactions for capturing CTCs. This system enables flexible binding of arbitrary capture antibodies tailored to different cancer markers [13].

Basic experiments and preliminary clinical studies have demonstrated that our newly developed CTC-Chip is more sensitive than CellSearch for detecting CTCs and enables molecular biological analysis of captured CTCs [14,15]. Identification of CTCs on a CTC-Chip involves fluorescence-based detection [16,17]. Currently, human operators manually identify CTCs by visually examining all the cells, which is labor intensive and requires experience. Subjective judgment may sometimes lead to false positives or negatives, underscoring the need for an automated CTC detection system based on image analysis.

Advancements in artificial intelligence (AI) have accelerated machine learning research [18,19]. Machine learning offers the advantages of objectivity, speed, and noise resistance, making it widely used in medical imaging. Among the classical machine learning algorithms, deep learning and convolutional neural networks (CNNs) have significantly contributed to medical imaging research [20,21,22]. Furthermore, recent studies have highlighted the effectiveness of deep learning techniques in detecting and classifying rare cells. For example, He et al. successfully improved the accuracy of CTC image recognition using machine learning algorithms [23]. Additionally, AI-based analysis methods focusing on rare CTCs have shown potential as novel diagnostic markers for predicting metastasis and evaluating treatment efficacy in patients with cancer [24,25].

**Figure 1:**
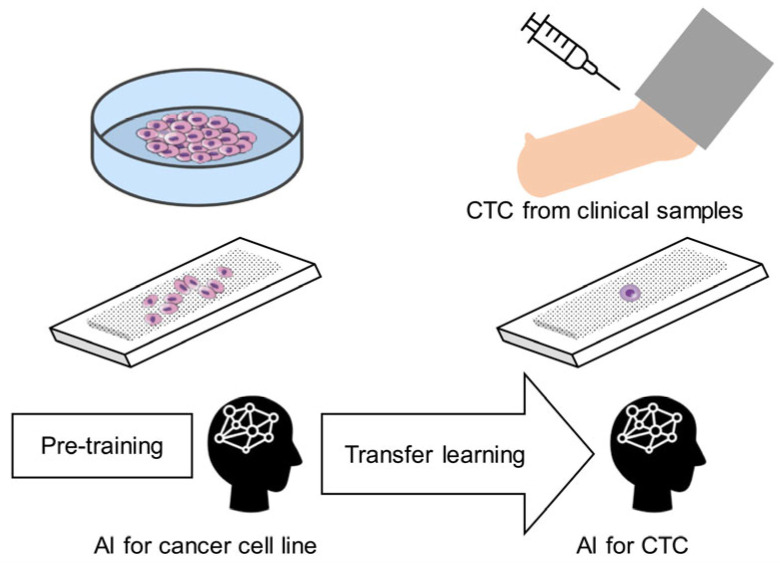
Overview of the circulating tumor cell (CTC) image recognition system workflow. Illustration of the process used to establish the highly accurate CTC recognition system, highlighting pre-training on lung cancer cell lines, image acquisition, segmentation, and transfer learning implementation. AI, artificial intelligence.

A CTC image recognition system requires a large CTC image dataset for training. However, CTCs are rare in clinical samples, making data collection difficult [26,27]. Instead, training on cancer cell lines, which broadly belong to the same category as CTCs, can reduce the burden of acquiring CTC image data, while allowing classifier development with fewer images across various cancer types. In this study, we aimed to develop a classifier for high-accuracy cell classification even with limited CTC image data by incorporating transfer learning with pre-training on cancer cell line images using an existing algorithm (Figure 1) [28]. We hypothesized that, compared to training exclusively on limited clinical CTC images, pre-training using images of lung cancer cell lines would significantly improve the classification accuracy of CTC images.

## 2. Materials and Methods

### 2.1. CTC-Chip Preparation

The CTC-Chip was prepared as described previously [14,15]. Initially, goat anti-mouse IgG (SouthernBiotech, Birmingham, AL, USA) was incubated overnight at 4 °C as the base, followed by incubation with mouse anti-human EpCAM antibody at 25 °C for 1 h (“EpCAM-Chip”).

### 2.2. Cell Lines

Three human lung cancer cell lines, PC9 (RCB4455, obtained from RIKEN BioResource Research Center, Kyoto, Japan), NCI-H441 (HTB-174, obtained from ATCC, Manassas, VA, USA), and A549 (CCL-185, obtained from ATCC), were used for pre-training. Cells were cultured in RPMI-1640 medium (Wako Pure Chemical Industries, Osaka, Japan) and supplemented with 10% fetal bovine serum (Thermo Fisher Scientific, Waltham, MA, USA) at 37 °C in an atmosphere of 5% CO_2_.

These cell lines were selected for their representative diversity of genetic and histological profiles commonly observed in lung cancer. Specifically, PC9 harbors *EGFR* mutations commonly associated with lung adenocarcinoma, A549 is characterized by *KRAS* mutations and serves as a standard adenocarcinoma model, while NCI-H441 represents another distinct adenocarcinoma subtype. The extensive application through research validation and suitability for fluorescence-based imaging make these cell lines ideal for establishing a robust and generalizable CTC classification system. Subsequently, the cell lines were suspended in phosphate-buffered saline (PBS) and introduced onto the CTC-Chip for analysis.

### 2.3. Clinical Samples

In total, 119 patients with lung cancer diagnosed at the Second Department of Surgery, University of Occupational and Environmental Health, between 2014 and 2020, were included. Peripheral blood samples were collected before treatment and processed using a CTC-Chip.

### 2.4. Fixation, Staining, and CTC Identification on the Chip

**Figure 2:**
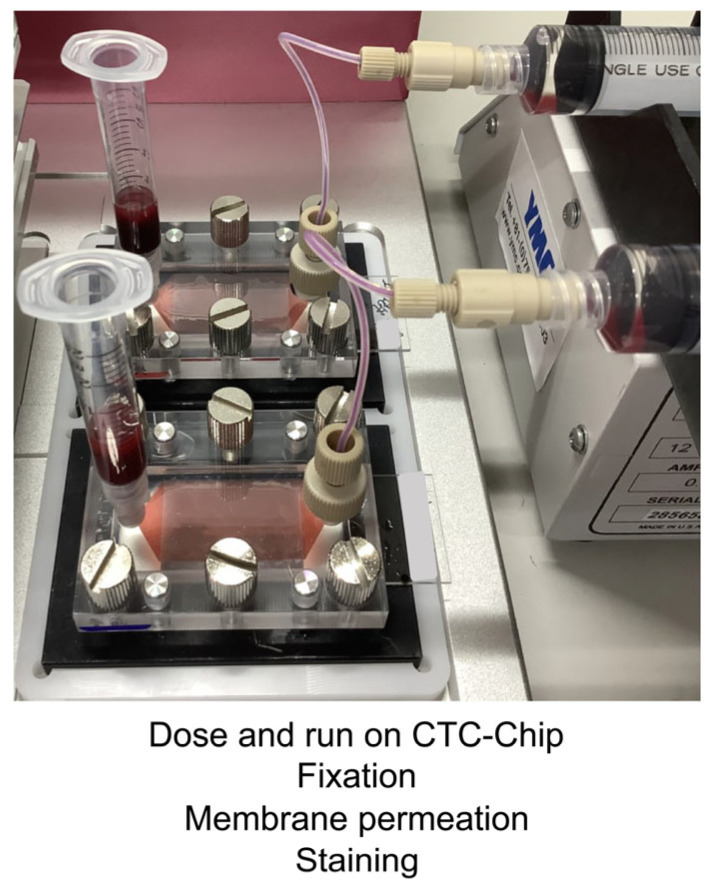
CTC identification and staining protocol on the CTC-Chip. Detailed workflow describing fixation, permeabilization, and fluorescence staining with Hoechst 33342, cytokeratin, and CD45 antibodies for distinguishing CTCs from leukocytes.

The samples were introduced to the EpCAM Chip and subjected to cell fixation, membrane permeabilization, and staining (Figure 2). Cells were fixed in 4% formalin, and 0.25% Triton was used for membrane permeabilization. Captured cells were incubated with primary antibodies, rabbit anti-cytokeratin (CK) antibody (ab9377; Abcam, Cambridge, UK), and rat anti-CD45 antibody (clone YTH24.5; Abcam), at 25 °C for 1 h. Secondary antibodies—Alexa Fluor 594 anti-rabbit IgG and Alexa Fluor 488 anti-rat IgG—were used for 30 min incubation at 25 °C. A fluorescence microscope (DMi8-S2G; Leica Microsystems, Wetzlar, Germany) was used for imaging. Cells with a round or oval morphology, Hoechst 33342-positive nuclei, CK-positive cytoplasm, and CD45-negative staining were visually identified as CTCs.

CTC identification in clinical samples was independently assessed by two researchers blinded to the clinical data. Discrepancies were resolved through a simultaneous review.

### 2.5. Processing of Captured Images Using AI and Classification by Computation

The cell classification process using the CNN-based algorithm is outlined below. Cell regions were extracted from microscopic images of the cell lines and clinical blood samples. The following morphological criteria were used for cell image extraction: Otsu’s method [29] was used to extract Hoechst-positive areas (blue components). Positional information of the cells for segmentation was obtained based on the blue component image. Using this positional information, the red and green components at the same location were extracted, and antibody-stained cell images were obtained by synthesizing each segmented component at the same location.

Next, brightness was analyzed to control the background information of the cell images. The brightness components of each fluorescent signal were combined into a single image. The images were subsequently input into a CNN-based classifier for cell evaluation.

**Figure 3:**
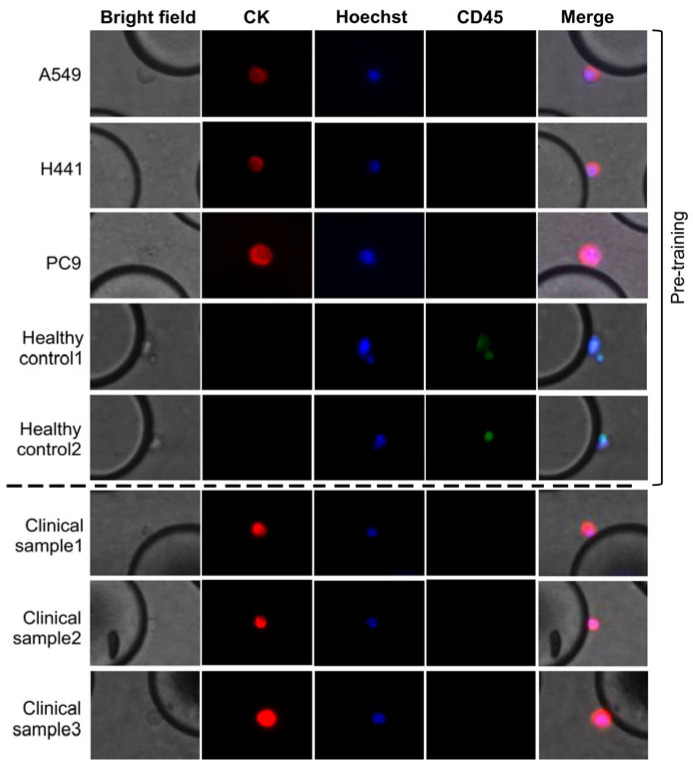
Representative microscopic images of captured cells on the CTC-Chip. (above the dotted line) Representative images of lung cancer cell lines (PC9, NCI-H441, and A549); (below the dotted line) clinical CTC images identified based on fluorescence staining: Hoechst-positive nuclei (blue), CK-positive cytoplasm (red), and CD45-negative (green). Cells with a combination of red and blue (i.e., pink) are defined as CTCs.

Specifically, lung cancer cell lines with PBS and clinical blood samples were processed on the chip, as described above. Images were acquired using a fluorescence microscope (DMi8-S2G; Leica Microsystems) at 10× magnification using an objective lens. The obtained images were processed using an algorithm developed in collaboration with the Department of Mechanical Intelligence and Systems Engineering at the University of Toyama. Representative cell images are presented in Figure 3.

All imaging data utilized in this study were obtained exclusively from our CTC-Chip platform and clinical samples. No external datasets, including the Kaggle database, were used.

### 2.6. Data Augmentation

We applied several standard data augmentation techniques during preprocessing to mitigate potential overfitting resulting from the limited dataset size. Specifically, training images underwent augmentation through random rotations (0–360°), horizontal and vertical flips, random scaling, adjustments to image brightness and contrast, and minor image translations. These preprocessing steps artificially increased dataset variability, thereby improving the robustness and generalizability of our CNN-based classification model.

### 2.7. Hardware and Software Environment Used for Computation

The hardware environment comprised the following:

CPU: Intel^®^ Core™ i9-10980XE CPU @ 3.00 GHz (Intel Corporation, Santa Clara, CA, USA)

Memory: 96 GB

GPU: NVIDIA GeForce RTX 3090 (Nvidia Corporation, Santa Clara, CA, USA)

V-RAM: 24 GB

The software for computation included:

Python Ver.3.6.13

CUDA Ver.10.1

OpenCV Ver.4.5.3.56

cuDNN Ver.7.6.5

TensorFlow Ver.2.6.0

Keras Ver.2.6.0

NumPy Ver.1.19.5

pandas Ver.1.1.5

openpyxl Ver.3.0.9

matplotlib Ver.3.3.4

scikit-learn Ver.0.24.2

seaborn Ver.0.11.2

shap Ver.0.40.0

### 2.8. Acquisition of Pre-Trained Models

#### 2.8.1. Data Conditions for Pre-Training

The cancer cell lines used in this study are presented in Table 1. Five types of cells were used as follows: three lung cancer cell lines and healthy cells obtained from the blood samples of two healthy individuals. The five cell types were classified into two groups, cancerous and healthy cells. The numbers of training data samples for each cell type are listed in Table 1.

#### 2.8.2. Addressing Data Imbalance During Pre-Training

In this study, the number of cancer cell lines used for pre-training was significantly lower than that of healthy cells, leading to an imbalance in the dataset. To resolve this issue, we adopted a method proposed in a previous study [30] that balances data within minibatches.

#### 2.8.3. Model Conditions for Pre-Training

**Figure 4:**
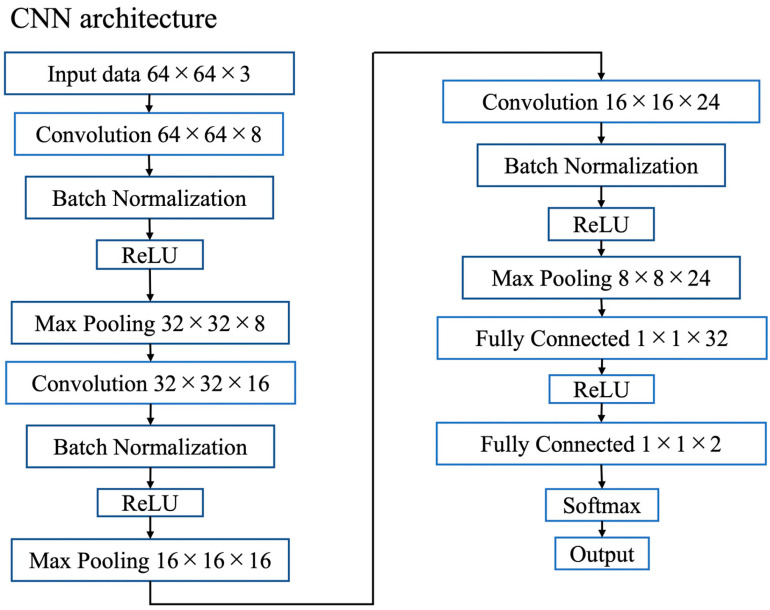
Convolutional neural network (CNN) architecture used in this study. Schematic illustration of the CNN layers, demonstrating the convolution, pooling, fully connected layers, and output classification for distinguishing CTCs from non-CTCs.

The hyperparameters and layer structures used for pre-training are described. The CNN layer structure used in this study is illustrated in Figure 4. Several models were prepared by varying the number of epochs and using minibatch creation methods during pre-training. These models were subjected to transfer learning to verify their accuracy and select the optimal model.

Table 2 presents the hyperparameters that remained unchanged and those that varied during pre-training. Varied hyperparameters included six epoch counts and two minibatch creation methods.

### 2.9. Evaluation of Transfer Learning

#### 2.9.1. Comparison of Classification Accuracy Between Models with and Without Transfer Learning (Using Clinical Data Only)

The effectiveness of transfer learning was evaluated to achieve high classification accuracy with a limited number of clinical images. First, the optimal pre-trained model was selected based on prior comparisons. Subsequently, the classification accuracy was compared between a transfer learning model and a model trained only on clinical data. The number of training images used for transfer learning varied, and the transition in classification accuracy was measured.

**Figure 5:**
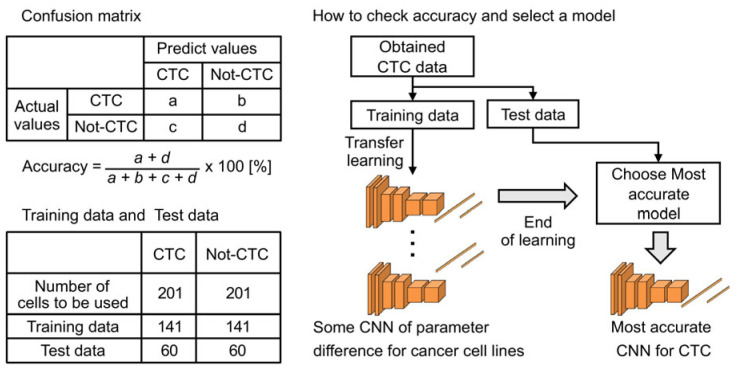
Transfer learning evaluation methodology. Schematic description of dataset division into training and test sets, detailing the evaluation procedure using repeated sampling (100 samples) to measure classification accuracy.

Accuracy Evaluation (Figure 5):

• χData Splitting: CTCs and non-CTCs obtained from clinical samples were divided into training and testing datasets. The model was trained using the training data, and its accuracy was evaluated using the test data. One sample comprised the entire process, from data splitting to accuracy evaluation.
• ϕEvaluation Method: Training and testing were repeated on 100 samples to assess the classification accuracy. The accuracy was used as an evaluation metric.

Parameter Conditions:

• αData: The numbers of CTCs and other cells obtained from the patient samples were divided into training and test datasets, which are shown in Table 1. A total of 201 CTCs were visually identified in clinical samples. Although non-CTCs were more abundant, 201 images were randomly selected to match the number of CTCs, thereby preventing data imbalance.
• ρHyperparameters: The CNN model structure and epoch count were adjusted during pre-training. The hyperparameters, including the learning rate, batch size, and dropout rate, were optimized using a systematic grid search, evaluating multiple epoch numbers and sampling methods to determine the most effective parameters based on validation accuracy.

#### 2.9.2. Comparison of Classification Accuracy Between Models with and Without Transfer Learning (Using Only Pre-Training)

The classification accuracies of the models using transfer learning and those trained only with pre-trained cancer cell lines were compared. We evaluated the significance of training CTCs through transfer learning by comparing the recognition accuracy of models trained solely on pre-trained cancer cell lines with that of models subjected to transfer learning.

Furthermore, the recognition accuracy of models trained only on pre-trained cancer cell lines was compared with that of models using different numbers of training images during transfer learning. This analysis aimed to determine the optimal number of training images required to improve the classification accuracy.

### 2.10. Statistical Analysis

Welch’s *t*-test was used to compare the CTC recognition accuracy between the pre-trained models and models using different numbers of training images ranging from 1 to 141 images. The statistical significance was assessed.

The effect of using very few training images in transfer learning was evaluated. CNN-based deep learning algorithms generally require large datasets. When training CNNs with a few images, overfitting may occur, resulting in reduced recognition accuracy [31,32]. Subsequently, Welch’s *t*-test was performed to compare the recognition accuracy between the pre-trained model (trained on cancer cell lines) and the transfer-learned model trained on only one image. Statistical significance was determined.

All analyses were conducted using SPSS MAC version 24.0. Statistical significance was set at *p* < 0.05.

## 3. Results and Discussion

### 3.1. Does Training on Cell Lines Actually Help?

Transfer Learning vs. No Transfer Learning (Clinical Images Only):

• Classification accuracy improved for all training sample sizes when transfer learning was performed.
• A statistically significant difference was observed between the two groups, particularly when the number of training samples was 19 or fewer.

**Figure 6:**
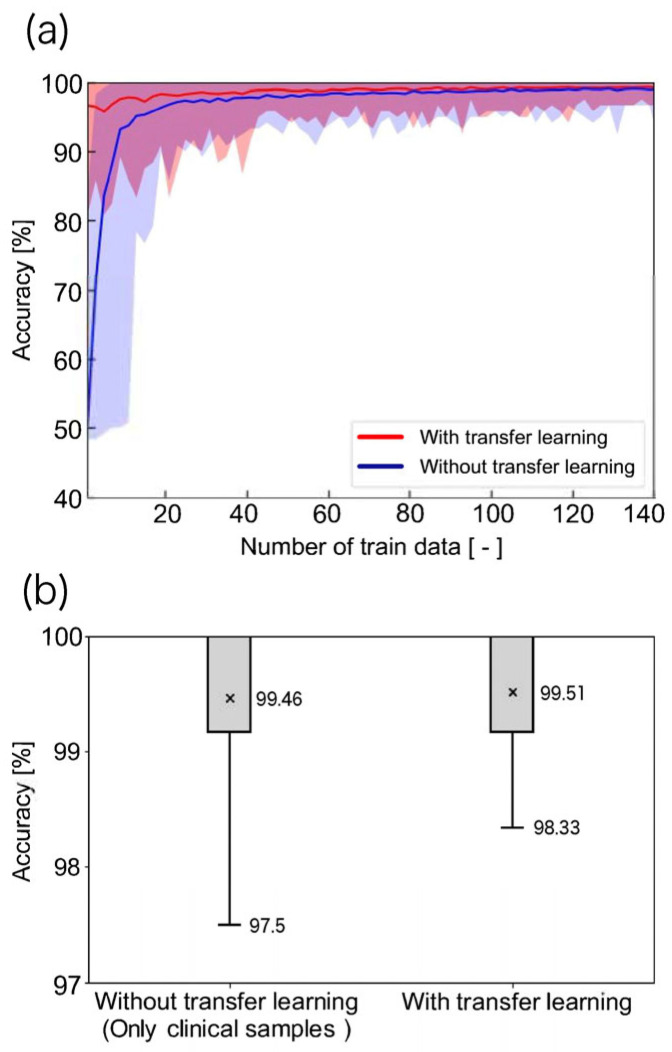
Impact of transfer learning on classification accuracy. (**a**) Comparison of accuracy between models with and without transfer learning across varying numbers of clinical training images. A significant improvement was observed with fewer than 20 training images (*p* < 0.05); (**b**) comparison of the accuracy at the maximum training image count (n = 141), indicating no negative impact of pre-training.

We compared the classification accuracy with and without transfer learning using a CNN model to evaluate the effect of varying the number of clinical training images during transfer learning. The trend of classification accuracy with varying training sample sizes is shown in Figure 6a. The classification accuracy with transfer learning was higher across all training sample sizes than those without transfer learning, indicating that transfer learning can achieve high-accuracy CTC recognition within the range of training sample sizes utilized in this study (up to 141 images).

Moreover, the effect of transfer learning was pronounced when the number of training samples was less than 20, with statistically significant differences observed for 19 or fewer samples (*p* < 0.05). This suggests that transfer learning is highly effective during the early stages of training when clinical images are limited. Thus, pre-training with cell lines is particularly beneficial when clinical image availability is low.

### 3.2. Does Training on Cell Lines Negatively Affect Performance?

The classification accuracy at the end of 141 training samples was compared between the models with and without transfer learning, as shown in Figure 6b:

• Minimum classification accuracy with transfer learning: 98.33%.
• Minimum classification accuracy without transfer learning: 97.5%.
• Accuracy improvement with transfer learning: 0.83%.

The average classification accuracy was as follows:

• With transfer learning: 99.51%.
• Without transfer learning: 99.46%.

No statistically significant difference was observed in the average classification accuracy, indicating that transfer learning did not degrade the performance.

### 3.3. Is Pre-Training on Cell Lines Alone Sufficient?

Transfer Learning vs. No Transfer Learning (Pre-training Only):

• Average recognition accuracy with pre-training only: 96.96%.
• Average recognition accuracy with transfer learning: 99.51%.
• Accuracy improvement with transfer learning: 2.55% (statistically significant).
• Minimum recognition accuracy improvement: from 84.16% to 96.67% (+12.51%).

Next, we compared the classification accuracy of a model trained only on pre-trained cell lines with that of a model subjected to transfer learning. The average recognition accuracy for the pre-training-only model was 96.96%, whereas that for the model subjected to transfer learning was 99.51%. This represents a statistically significant improvement of 2.55%.

**Figure 7:**
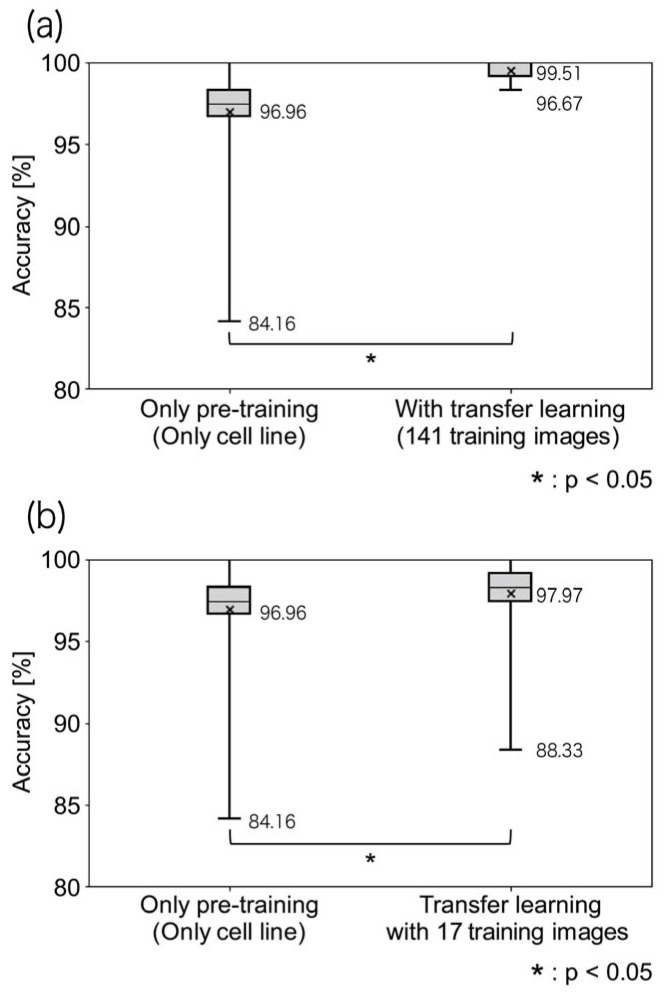
Importance of clinical imaging data in transfer learning. (**a**) Comparison of classification accuracy between models trained only with pre-trained cell lines versus transfer learning with clinical images. Results demonstrated a significant improvement in accuracy with transfer learning; (**b**) analysis showing the minimum number of clinical images required (≥17) for a statistically significant improvement compared to pre-training alone (* *p* < 0.05).

When comparing the minimum recognition accuracy, the pre-training-only model achieved 84.16%, whereas the transfer learning model achieved 96.67%, demonstrating an improvement of 12.51%. These results confirm the importance of training on CTCs along with the pre-training of cancer cell lines (Figure 7a). Furthermore, these findings suggest that the characteristics of cancer cell lines and CTCs are not identical.

### 3.4. Effect of Training Sample Size

A statistically significant difference was observed when the number of clinical training images exceeded 17 compared to that obtained upon pre-training alone. Next, we examined the minimum number of clinical training images required to achieve a statistically significant improvement over pre-training alone. The average accuracy increased from 96.96% (pre-training only) to 99.51% (with transfer learning). The results are shown in Figure 7b. Compared to pre-training alone, the minimum accuracy increased from 84.16% to 96.67% when 17 or more clinical training images were used, which is a statistically significant improvement.

Recent studies utilizing AI-based methods for CTC detection have demonstrated significant advancements in the accuracy and efficiency of cell classification systems. Table 3 compares recent methodologies and their reported accuracies in similar AI-driven CTC detection research, highlighting the superiority of our transfer learning approach that integrates cell line images and limited clinical data.

Compared to conventional manual classification approaches, our AI-based method significantly enhanced objectivity, reduced human error, and improved processing speed. Traditional manual techniques require extensive expertise and are labor intensive, leading to potential subjectivity and inconsistent results. By leveraging CNN-based algorithms, our method enables faster, scalable, and more reproducible analysis, highlighting a clear advantage over conventional image processing techniques.

Additionally, our approach—based on pre-training and transfer learning techniques—can significantly improve real-time clinical image processing. By initially training CNNs with extensive cell line image data and subsequently fine-tuning with limited clinical CTC images, our model efficiently adapts to clinical scenarios. This hybrid approach ensures rapid, accurate, and consistent detection of CTCs in real-time clinical settings, substantially enhancing the practical applicability of CTC analysis.

In this study, we successfully improved the classification accuracy of CTCs in patients with lung cancer by performing pre-training using lung cancer cell lines. This approach is particularly effective in environments with limited clinical samples because it enables high-accuracy classification using only a small amount of clinical data, demonstrating its practical applicability in clinical settings.

In real-world clinical practice, constructing a universal classifier is highly challenging because of the diversity of image data, which includes variations in cancer types, staining methods, and imaging techniques. In particular, because CTCs are rare in clinical samples, the number of captured CTCs may be insufficient, leading to a significant decline in the classifier performance and complicating practical applications. However, our study revealed that pre-training with cell line data enabled high accuracy in the initial stages, making it a viable solution.

Nevertheless, our study indicates that training with cell lines alone is insufficient. A key finding was that combining training with both cell lines and clinical CTCs (mixed training) further improved classifier accuracy and generalizability. This approach provides flexibility in handling diverse clinical datasets, allowing the classifier to adapt even when the cancer types or staining methods differ.

Additionally, our results confirmed that, as the volume of the clinical sample data increased, the classification accuracy stabilized. This finding suggests that pre-training with cell lines does not negatively impact learning using clinical data. Instead, pre-training enhances the initial performance of the model, allowing for more efficient and accurate optimization when additional clinical data are introduced.

### 3.5. Biological Differences Between Cell Lines and Clinical Lung Cancer CTCs

Lung cancer cell lines provide significant advantages for training image recognition systems, including reproducibility and ease of handling; however, it is important to acknowledge their biological differences compared to clinical lung cancer-derived CTCs. Cell lines generally consist of cancer cells adapted to in vitro conditions, often selected for their rapid proliferation and stable growth under artificial culture conditions. In contrast, clinical CTCs directly obtained from patient blood samples exhibit the biological heterogeneity and variability inherent to cancer cells circulating in vivo. This variability is influenced by patient-specific tumor microenvironments, immune interactions, therapeutic regimens, and metastatic potential [1,27].

Consequently, while pre-training using lung cancer cell lines significantly enhances the initial performance of image recognition systems, exclusive training on these cell lines may inadequately represent the complexity and diversity of clinical CTCs. Thus, combining pre-training on cell lines with subsequent training on clinical CTC data is essential for maximizing the accuracy and generalizability of the CTC detection system.

### 3.6. Limitations

Despite these important findings, we acknowledge that this study has certain limitations:

1. Bias in cell line selection: This study focused on pre-training using lung cancer cell lines; however, further validation is required to determine whether the same approach applies to other cancer types and cell lines. For example, verifying the effectiveness of this method in CTC detection systems for breast or colorectal cancer would help establish its generalizability.
2. Diversity of clinical sample data: The clinical samples used in this study were collected under limited conditions that do not fully reflect the diversity encountered in real-world clinical settings. It is necessary to investigate how differences in patient backgrounds (e.g., age, sex, and disease stage) and variations in sample collection protocols across different institutions may have affected the results.
3. Limited dataset size: Although this study demonstrated the effectiveness of pre-training and transfer learning, a larger dataset is needed for further validation. Particularly, multi-institutional collaboration is crucial for collecting diverse patient samples and conducting comprehensive evaluations to enhance the clinical applicability of the model.
4. Impact on the overall CTC detection process: This study primarily focused on the effectiveness of transfer learning in image classification but did not evaluate its impact on the entire CTC detection pipeline, including the capture, staining, and data acquisition processes. Therefore, a more comprehensive investigation is required to assess these aspects.
5. Evaluation of other deep learning architectures: Future studies should explore and compare alternative deep learning architectures, such as Vision Transformer or hybrid models (CNN + LSTM), to potentially improve classification accuracy and yield new insights into CTC recognition. Comparative studies will elucidate the optimal architecture suitable for specific imaging tasks and conditions.

### 3.7. Future Directions

It is necessary to investigate whether mixed training using cell lines and clinical data can be applied to other cancer types and diagnostic workflows. Specifically, it is essential to develop model architecture and data augmentation techniques to accommodate different cancer types and new staining protocols. Additionally, conducting multi-institutional studies and long-term follow-ups is crucial to further validate the generalizability and clinical applicability of the model. Furthermore, to optimize the CTC detection process, research should be conducted in parallel with capture technologies, staining protocols, and the standardization of imaging techniques. Overcoming these challenges is expected to contribute to broader clinical applications of CTC detection technology.

A recent study [33] demonstrated the effective use of single-cell transcriptomic data to characterize CTCs. Such single-cell data strategies are complementary to our imaging-based classification approach, and their integration may enhance the understanding of CTC heterogeneity at a molecular level. Future research should explore combining AI-driven image classification with single-cell multi-omics techniques, potentially providing a more comprehensive and detailed characterization of CTCs.

### 3.8. Direct Comparison with Expert Pathologist Evaluation

As previously reported by our co-author in a related study of esophageal cancer [28], future research should focus on a direct comparison between AI-based detection and manual detection conducted by experienced pathologists. Clearly defining sensitivity, specificity, and other relevant metrics will further validate the clinical applicability of our CNN-based CTC detection system for lung cancer.

This study demonstrates that pre-training using lung cancer cell lines significantly improves the classification accuracy of CTC images in clinical samples. In particular, high-precision CTC image recognition can be achieved even when clinical imaging data are limited. This finding presents a new approach that efficiently utilizes rare CTC data to enhance diagnostic accuracy in clinical settings.

Pre-training with cell lines can serve as a robust foundation for rapidly developing image recognition systems for screening new cancer types, staining methods, or imaging protocols. This flexibility significantly expands the applicability of the CTC detection technology for cancer diagnosis and treatment monitoring, making it an important technique for future medical image analyses.

## 4. Conclusions

Our approach enhances the efficiency and feasibility of CTC detection technology in clinical practice. Compared with conventional methods, it reduces the need for collecting large amounts of clinical imaging data while enabling rapid and high-accuracy CTC classification at the early stages of diagnosis. This technology can contribute to early cancer detection and real-time treatment effectiveness monitoring, ultimately improving patient prognosis.

It is crucial to explore the applicability of this method to other cancer types and new diagnostic processes to further enhance the versatility and clinical utility of CTC detection technology. The findings of this study represent an important step toward the development of personalized medicine and more efficient cancer treatments. 

## Figures and Tables

**Table 1:** Model of data conditions during pre-training.

Types of Cancer Cell Lines					
	**Cancer Cell Lines**			**Healthy Controls**	
Cell types	A549	H441	PC9	Healthy control 1	Healthy control 2
Number of samples obtained	4289	1555	3412	21,648	9287
**Number of pre-training samples**					
	**Cancer cell lines**			**Healthy controls**	
Training samples	9106			30,785	

**Table 2:** Hyperparameters during pre-training.

Unchanged Hyperparameters	
Iteration	20
Learning	0.001
Optimizer	SGD (momentum: 0.9)
Loss function	Cross-entropy
**Changed hyperparameters**	
Epoch	2, 6, 12, 25, 50, 100, and 300
Sampling method	Balanced minibatch, All data

SGD, stochastic gradient descent.

**Table 3:** Comparison of recent studies utilizing AI-based CTC detection methods.

Author (Year)	Methodology	Cancer Type	Training Data	Accuracy
He et al. (2020) [23]	Machine learning	General cancers	Clinical images	92.5%
Zeune et al. (2020) [20]	Deep learning	Various cancers	Clinical images	93.8%
Wang et al. (2020) [21]	Label-free detection and deep learning	General cancers	Clinical images	94.3%
Akashi et al. (2023) [28]	AI-based detection	Esophageal cancer	Clinical images	95.0%
Matsumiya et al. (Present study)	Transfer learning CNN	Lung cancer	Cell lines + Clinical images	99.51%

## Data Availability

The original contributions presented in this study are included in the article. Further inquiries can be directed at the corresponding author.

## Abbreviations

The following abbreviations are used in this manuscript:

AI	Artificial Intelligence
CK	Cytokeratin
CNN	Convolutional neural networks
CTC	Circulating Tumor Cell
CUDA	Compute Unified Device Architecture
IgC	Immunoglobulin G
RPMI	Roswell Park Memorial Institute (medium)
SGD	Stochastic Gradient Descent
V-RAM	Video Random Access Memory
